# Effect of adolescent female fertility and healthcare spending on maternal and neonatal mortality in low resource setting of South Asia

**DOI:** 10.1186/s13561-022-00395-7

**Published:** 2022-09-17

**Authors:** Shongkour Roy, Tanjina Khatun

**Affiliations:** 1 Population Council, Dhaka 1212, Bangladesh; 2 Mirpur Government Bangla College, University of Dhaka, Dhaka 1216, Bangladesh

**Keywords:** Neonatal mortality, Maternal mortality, Adolescent female fertility, Random effect model, Healthcare spending, Multiple imputation

## Abstract

**Background:**

Maternal and neonatal mortality is high in South Asia. Recent studies have identified factors such as adolescent female fertility, healthcare spending is reducing maternal and neonatal mortality. The objective of this study is to examine the effect of adolescent female fertility and healthcare spending on maternal and neonatal mortality in South Asian countries.

**Methods:**

A retrospective panel study design was used, a total of 8 South Asian countries (Afghanistan, Bangladesh, Bhutan, India, Maldives, Nepal, Pakistan, and Sri Lanka) data from World development indicator 1990–2020 considered for analysis. Descriptive statistical method was used for summary. The effect of adolescent female fertility and healthcare spending on maternal and neonatal mortality were analysed using fixed and random effect regression with multiple imputation.

**Findings:**

Adolescent female fertility, maternal, and neonatal mortality is very high in the aforementioned countries, and considerably varies among countries. A significant relationship between the maternal mortality and healthcare spending, neonatal mortality and adolescent female fertility was observed. We found neonatal and maternal mortality are more likely to decrease depends on healthcare spending. Healthcare spending has a significantly negative effect on neonatal mortality (− 0.182, 95% CI: [− 0.295 to −.069]; *P*-value < 0.01) and maternal mortality (− 0.169, 95% CI: [− 0.243 to − 0.028]; *P*-value < 0.05). A change in 1 % increases in healthcare spending should decrease by 0.182 neonatal mortality per 1000 live births and maternal mortality by 0.169 per 100,000 live births.

**Conclusions:**

In south Asian countries, increasing healthcare spending and decreasing adolescent female fertility may contribute to reduce maternal and neonatal mortality. In addition, number of service providers such as physicians supplied contributed to the decline of neonatal mortality. These findings have important implications for future improvement of healthcare spending in maternal and neonatal health programs.

**Supplementary Information:**

The online version contains supplementary material available at 10.1186/s13561-022-00395-7.

## Introduction

Maternal and neonatal mortality in Asian countries has been decreasing over time [1]. Healthcare spending strongly influences both neonatal and maternal mortality [2]. Healthcare spending and adolescent female fertility are key policy instruments that are expected to decrease neonatal and maternal mortality. A global estimate found that each year, 17 million adolescent girls give birth, which allocates for 11.0% of all births worldwide. The most of these births occurred in low- and middle-income countries [3], and adolescent female fertility is the top cause of mortality among girls aged 15–19 [4]. Neonatal and maternal mortality could be decreased using health capital collection and health improvement through longevity and increased healthcare spending. Besides, an increase in healthcare spending, the government’s expanding cost on female education, and taking a step from lower female adolescent fertility can improve maternal and neonatal health. Maruthappu has examined the association between government healthcare spending and maternal mortality in European Union countries [5]. This article used longitudinal data between the period 1981 to 2010 and found that reductions in government healthcare expenditure were significantly associated with increased maternal mortality.

The study of healthcare spending on neonatal and maternal mortality in developed countries has been growing fast over the last decade. However, a few studies have been conducted in developing countries that look like actors in healthcare spending and adolescent female fertility [6, 7].

The main purpose of this article is to examine the

(i) Variation in adolescent female fertility, healthcare spending, neonatal and maternal mortality across 8 South Asian countries; and (ii) Do adolescent female fertility rates and healthcare spending on neonatal and maternal mortality have significant effects on the selected South Asian countries?

In doing so, a fixed and random effects model was developed among the selected countries, which covered the healthcare spending on neonatal and maternal mortality. Specifically, an attempt is being made to estimate the effects of healthcare spending, physicians, adult female literacy rate, and adolescent female fertility on neonatal and maternal mortality in South Asian countries using a panel data set comprising 31 observations for each of the eight individual countries, thereby providing 248 observations to carry out robust empirical examinations.

The definition of healthcare expenditure is given by the total sum of expenditure on healthcare functions, as, for example, total healthcare services, medical goods, prevention and public health services, health insurance, and public healthcare expenditures are shared by the government [8]. Some panel data studies have examined the effect of governance measures on health outcomes [9, 10]. This emerging literature has examined the role of the governance environment in the effectiveness of health expenditure. Rajkumar and Swaroop have investigated the relationship between public health expenditure and life expectancy and literacy rates [11]. They used three-year panel data (1990, 1997, and 2003) covering 91 developed and developing countries to estimate how healthcare expenditure affected the relationship.

The literature on the relationship between healthcare spending and neonatal mortality has not yet come to a consensus in South Asia. This problem arises because of doubts about the estimated effects of healthcare spending. The uncertainty could be attributed to the use of different and inconsistent data, accounting for or not accounting for the endogeneity of healthcare expenditure, and unobserved heterogeneity in the methods of estimation. We also investigated the possibility that maternal health seeking might act as a mediator between physicians and neonatal mortality endpoints.

## Methods

### Study design and population

The panel retrospective study included all maternal and neonatal mortality, which had been presented in numbers in South Asian countries between 1990 to 2020. This study was used as the WHO definition of maternal and neonatal mortality.

### Data collection and variables

The subjects of this study have cover 8 South Asian countries. The data was collected by country and year from the World Development Indicator (WDI). Healthcare spending was expressed as a percentage of GDP and expressed as per capita international dollars. They were converted into real terms using inflation data from the WDI.

Healthcare spending is a health function that enables the purchase of health goods and services. Healthcare spending is likely to influence the quality of health services and improve neonatal and maternal health. The outcome variables are neonatal mortality per 1000 live births (NM) and maternal mortality per 100,000 live births (MM). The independent variables included healthcare spending per capita (HCEP), physicians per 1000 people (P), the adult female literacy rate in percentage (AFLR), and adolescent fertility rate per 1000 women aged 15–49 (AFR). The detailed definition and measures are in Table S1.

### Statistical analysis

The empirical model was used to investigate the effect of healthcare spending on neonatal and maternal mortality. All readers are interested can also see a different study on healthcare spending [12–15].

The model begins with neonatal and maternal mortality specified in a panel form as follows:

1 $${y}_{nt}={X}_{nt}\beta +{\epsilon}_t,t=1,\dots, T$$

2 $${\epsilon}_t=\xi Z+\vartheta$$

where *y*_*nt*_ is a dependent variable indicating neonatal mortality (*NM*_*nt*_) and maternal mortality (*MM*_*nt*_) in south Asian country n (*n* = 1,2, ….,8) at time t (t = 1990, ….,2020), and are continuous variables that takes value of neonatal and maternal mortality rate. X is a vector of independent variables indicating healthcare expenditure (*HEPC*_*nt*_), physicians (*P*_*nt*_), adult female literacy rate (*ALFR*_*nt*_), and adolescent female fertility rate (*AFR*_*nt*_) south Asian country n (*n* = 1,2, ….,8) at time t (t = 1990,….,2020), and all independent variables are continuous. The constant β is a vector of coefficients. *ϵ*_*t*_ is a vector of random error terms and eq. (1) decomposing the error process into a summation of two components: the time variant and remainder error process. The error term is spatially correlated with the spatial weight matrix Z and has spatial autocorrelation parameter *ξ* in eq. 2.

Let us consider that the empirical model for neonatal and maternal mortality is specified for the purpose of study as follows-

3 $${lnNM}_{nt}=\alpha_n+\alpha_1{lnHEPC}_{nt}+\alpha_2P_{nt}+\alpha_3{lnAFLR}_{nt}+\alpha_4{lnAFR}_{nt}+\varepsilon_{nt}$$

4 $${lnMM}_{nt}=\beta_n+\beta_1{lnHEPC}_{nt}+\beta_2P_{nt}+\beta_3{lnAFLR}_{nt}+\beta_4{lnAFR}_{nt}+\varepsilon_{nt}$$

Where, *lnNM*_*nt*_ *NMandMM* and *lnMM*_*nt*_ are dependent variables of neonatal and maternal mortality, and the remaining variables indicating natural-log of healthcare expenditure (*lnHEPC*_*nt*_), physicians (*P*_*nt*_), natural-log of adult female literacy rate (*lnAFLR*_*nt*_), and natural-log of adolescent female fertility rate (*lnAFR*_*nt*_) are independent, *n* refers to Asia countries (*n* = 1,2,….,8), *t* refers to time (t = 1990,….,2020), *α*_1_, *α*_2_, *α*_3,_ *and α*_4_ for neonatal mortality, and *β*_1_, *β*_2_, *β*_3,_ *and β*_4_ for maternal mortality are corresponding unknown parameters to be estimated. *α*_*n*_ and *β*_*n*_ is the time invariant and captures country-specific effect that was not included in the model and random error ${\varepsilon}_{nt}\sim N\left(0,{\sigma}_{\varepsilon t}^2\right)$. In Eqs. (3 and 4) it is hypothesized that the effects of HEPC, P, and AFLR would be negative and AFR would be positive.

The summary and correlation matrix were analysed. The fixed effects (FE) and random effects (RE) regressions were estimated using the multiple imputation method. For the parameters of panel data, one can choose an FE or RE model. Another way to select FE and RE models is by running the Hausman test, which provides evidence of the existence of correlations between individual effects and the regressors [16].

The choice of FE and RE depends on the Hausman tests statistical significance. When it is significant, the FE model can be used, otherwise the RE [16]. Moreover, to test the robustness of the coefficients, neonatal and maternal mortality have been regressed against healthcare spending, physicians, adult female literacy rates, and adolescent female fertility rate, and each of the countries was eliminated one by one. All these were estimated by using Stata software version 17.0 (Stata Corp LP, Lakeway Drive College Station, Texas, USA).

## Results

**Fig. 1:**
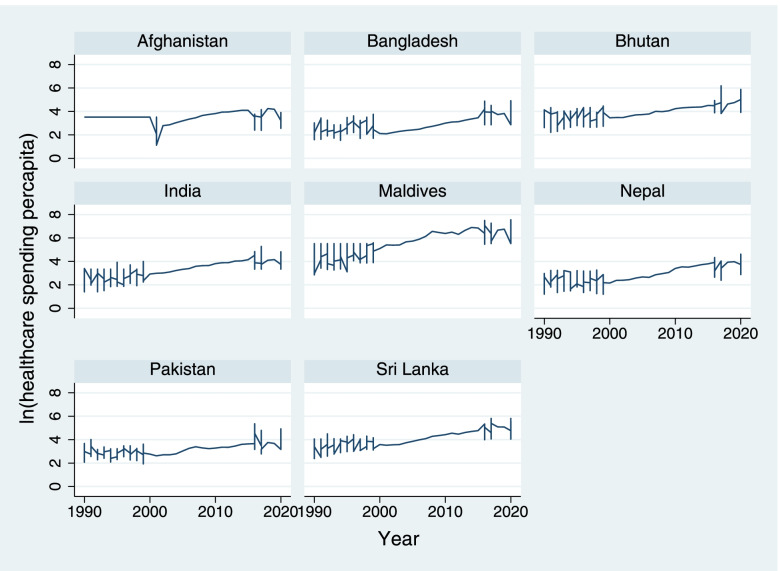
Variation of healthcare spending by Country (1990–2020)

Variations of the summary measure and correlation matrix of the selected variables are presented in Tables 1 and 2. The variation of healthcare spending by year in 8 South Asian countries is shown in Fig. 1. The correlation matrix provided the correct sign and supported for the hypothesis. As expected, the NM and MM are negatively related to HEPC, P, AFLR, and positively related to AFR.

**Table 1:** Variation of summary measure between 8 South Asian Countries (1990 to 2000)

Variables	Summary measure	Country Code								
		AFG	BGD	BTN	IND	LKA	MDV	NPL	PAK	Total
lnMM	Mean	8.80	8.87	4.00	10.51	4.86	2.70	7.70	9.11	7.05
	SD	1.02	1.58	0.93	2.21	1.06	1.40	1.46	1.29	3.00
	Min	0.48	0.21	−0.21	2.79	−0.63	−2.68	0.01	3.24	−2.68
	Max	12.33	10.55	7.94	11.93	7.64	10.95	10.64	14.08	14.08
lnNM	Mean	10.88	11.66	6.04	13.81	7.90	4.61	10.01	12.48	9.64
	SD	0.12	0.49	0.43	0.36	0.36	0.85	0.47	0.07	3.03
	Min	10.66	10.83	5.28	13.10	7.18	3.33	9.16	12.39	3.33
	Max	11.03	12.39	6.76	14.27	8.44	5.96	10.66	12.60	14.27
lnHEPC	Mean	3.53	2.87	3.94	3.35	4.07	5.53	2.89	3.15	3.68
	SD	0.58	0.62	0.59	0.68	0.64	1.05	0.68	0.49	1.08
	Min	1.48	1.53	2.62	1.40	2.48	3.11	1.19	2.07	1.19
	Max	4.25	4.92	6.19	5.24	5.81	7.56	4.22	4.92	7.56
p	Mean	0.23	0.32	0.34	0.65	0.56	1.11	0.23	0.68	0.52
	SD	0.19	0.24	0.33	0.29	0.33	0.77	0.34	0.23	0.48
	Min	−0.82	−0.44	−0.47	−0.49	−0.06	−0.41	−1.31	− 0.13	−1.31
	Max	0.93	1.17	1.91	1.70	1.66	3.61	1.11	1.47	3.61
lnAFLR	Mean	3.38	3.73	4.01	3.71	4.27	4.32	3.65	3.68	3.87
	SD	0.40	0.40	0.43	0.39	0.43	0.43	0.47	0.30	0.51
	Min	2.23	2.68	2.73	2.72	2.91	2.43	2.64	2.69	2.23
	Max	4.24	4.56	5.25	4.63	5.18	5.35	5.37	4.69	5.37
lnAFR	Mean	4.76	4.59	3.95	3.86	3.15	3.13	4.52	3.91	3.99
	SD	0.34	0.34	0.60	0.58	0.24	0.94	0.31	0.28	0.77
	Min	3.90	2.91	2.67	2.39	2.32	1.70	3.33	2.78	1.70
	Max	5.11	5.04	4.66	4.59	3.87	4.87	4.88	4.45	5.11

**Table 2:** Correlation matrix between selected variables with neonatal and maternal mortality

Neonatal mortality						Maternal mortality					
Variables	lnNM	lnHEPC	P	lnAFLR	lnAFR	Variables	lnMM	lnHEPC	P	lnAFLR	lnAFR
lnNM	1					lnMM	1				
lnHEPC	−0.6885***	1				lnHEPC	−0.707***	1			
P	−0.273***	0.672***	1			p	−0.288***	0.672***	1		
lnAFLR	−0.520***	0.583***	0.365***	1		lnAFLR	−0.587***	0.583***	0.365***	1	
lnAFR	0.493***	−0.803***	−0.693***	− 0.610***	1	lnAFR	0.561***	−0.803***	−0.693***	− 0.6100***	1

Inferences: *** *P* < 0.001; ** *P* < 0.010; *P < 0.05

The modelling results with the fixed and random effects model are reported in Table 3. The results of lnHEPC were statistically significant for both models (Table 3). The finding indicates that one unit increase in the lnHEPC brings about a 0.178 decrease in neonatal mortality per 1000 live births in the fixed effect model and a 0.182 decrease in neonatal mortality per 1000 live births when the random effect model was considered. A Hausman statistic implied that the random effects model was the best fit for the selected variables in the neonatal mortality panel data.

**Table 3:** Estimated effects of healthcare spending on Neonatal and Maternal mortality

	Dependent variable: Neonatal mortality		Dependent variable: Maternal mortality	
Variables	Fixed-effects	Random-effects	Fixed-effects	Random-effects
Intercept	8.796*** (16.74) < 0.525> [7.717 to 9.875]	8.863*** (12.06) < 0.734> [7.412 to 10.314]	10.083* (2.31) <.435> [1.5 to 19.61]	5.132***(4.13) < 0.843> [3.140to 6.018]
lnHEPC	−0.178** (−3.23) < 0.055> [−.291 to −.064]	−0.182** (− 3.26) < 0.056> [− 0.295 to −.069]	− 0.610 (− 1.18) < 0.515> [− 1.758 to 0.537]	−0.169*(− 2.83) < 0.93> [− 0.243 to − 0.028]
P	−0.156 (−2.61) < 0.059> [−.277 to −.035]	−.153 (− 2.47) < 0.062> [− 0.276 to −.030]	0.431** (3.60) < 0.092> [.188 to 0.531]	0.231**(3.45) < 0.084> [0.095 to 0.379]
lnAFLR	− 0.103* (− 1.47) < 0.069> [− 0.247 to −.041]	− 0.147 (− 1.46) < 0.071> [− 0.249 to − 0.040]	−0.194***(− 4.033.) < 0.057> [− 0.294 to − 0.00]	− 0.189***(− 3.86) < 0.053> [− 0.28 to − 0.080]
lnAFR	0.497*** (7.32) < 0.067> [0.359 to 0.635]	0.495*** (7.11) < 0.069> [0.355 to 0.634]	0.899***(.928) < 0.193> [0.543to 0.978]	0.889***(8.98) < 0.012> [0.680 to 0.946]
Hausman test (*P*-value)	15.3 (0.0.093)		4.52 (0.321)	
F-test (*p*-value)	119.79(< 0.001)	113.86(< 0.001)	140.7(< 0.001)	136.9(< 0.001)

Inferences: *** *P* < 0.001; ** *P* < 0.010; *P < 0.050. t- values are parentheses, standard errors are in Second brackets, and 95% confidence intervals are in third brackets

Similarly, one unit increase in the lnHEPC brings about a 0.610 decrease in maternal mortality per 100,000 live births in the fixed effects model and a 0.169 decrease in maternal mortality per 100,000 live births when the random effects model was considered. A Hausman statistic also implied that the random effects model was the best fit for the selected variables in the maternal mortality panel data.

On the other hand, one unit percentage change in adolescent female fertility rate carried a significant increase in neonatal mortality of 0.495 per 1000 live births (95% CI 0.355 to 0.634; *p* < 0.001) and maternal mortality of 0.889 per 100,000 live births (95%CI 0.680 to 0.946; *p* < 0.001) in the random effects model in South Asian countries. The results for testing the robustness of the coefficients were presented in Tables 4 and 5. Neonatal, and maternal mortality have been regressed against healthcare spending, and adult female literacy rate variables, and each of the countries has been eliminated one by one. The random effect model showed that lnHEPC and lnAFLR have negative and significant effects, and lnAFR has a positive and significant effect on neonatal and maternal mortality in all models.

**Table 4:** The random effects of HEPC on Neonatal mortality

Countries	Estimated equations	F-test	*p*-value
8 countries	lnNM = 8.863*** -0.183**lnHEPC-0.153P-0.104***lnAFLR + 0.495***lnAFR	113.86	< 0.001
Without Afghanistan	lnNM = 8.933***-0.215**lnHEPC-0.137P-0.107**lnAFLR + 0.483*** lnAFR	108.15	< 0.001
Without Bangladesh	lnNM = 8.359***-0.169***lnHEPC-0.148P-0.059*lnAFLR + 0.511*** lnAFR	115.36	< 0.001
Without Bhutan	lnNM = 9.468***-0.18**lnHEPC-0.168P-0.109*lnAFLR + 0.484***lnAFR	99.51	< 0.001
Without India	lnNM = 8.111***-0.179*lnHEPC-0.1361P-0.136*lnAFLR + 0.541*** lnAFR	104.70	< 0.001
Without Maldives	lnNM = 10.179***-0.178lnHEPC + 0.135P-0.191**lnAFLR + 0.393*** lnAFR	64.0	< 0.001
Without Nepal	lnNM = 8.641***-0.161*lnHEPC-0.160P-0.0.067***lnAFLR + 0.502*** lnAFR	98.14	< 0.001
Without Sri Lanka	lnNM = 8.802***-0.164**lnHEPC-0.141P-0.092lnAFLR + 0.524*** lnAFR	113.64	< 0.001
Without Pakistan	lnNM = 8.659***-0.150**lnHEPC-0.150P-0.123*lnAFLR + 0.488*** lnAFR	106.77	< 0.001

Inferences: *** *P* < 0.001; ** *P* < 0.010; *P < 0.050

**Table 5:** The random effects of HEPC on Maternal mortality

Countries	Estimated equations	F-test	*p*-value
8 countries	lnMM = 5.132*** -0.169*lnHEPC + 0.231**P-0.189****lnAFLR + 0.889***lnAFR	136.9	< 0.001
Without Afghanistan	lnMM = 5.432***-0.502**lnHEPC + 0.342**P-0.192***lnAFLR + 0.731*** lnAFR	121.8	< 0.001
Without Bangladesh	lnMM = 4.102***-0.732*lnHEPC + 0.541**P-0.191**lnAFLR + 0.942*** lnAFR	134.05	< 0.001
Without Bhutan	lnMM = 5.012***-0.453*lnHEPC + 0.402**P-0.230***lnAFLR + 0.534***lnAFR	108.3	< 0.001
Without India	lnMM = 4.49***-0.173*lnHEPC + 0.320**P-0.143***lnAFLR + 0.978*** lnAFR	98.2	< 0.001
Without Maldives	lnMM = 5.352***-0.387*lnHEPC + 0.160P-0.154**lnAFLR + 0.943*** lnAFR	80.3	< 0.001
Without Nepal	lnMM = 4.80***-0.153*lnHEPC + 0.180**P-0.152**lnAFLR + 0.737*** lnAFR	131.1	< 0.001
Without Sri Lanka	lnMM = 5.041***-0.162*lnHEPC + 0.153**P-0.193**lnAFLR + 0.804*** lnAFR	113.9	< 0.001
Without Pakistan	lnMM = 4.12***-0.104lnHEPC + 0.184**P-0.104**lnAFLR + 0.908*** lnAFR	120.5	< 0.001

Inferences: *** *P* < 0.001; ** *P* < 0.010; *P < 0.050

## Discussion

The findings of study, which used retrospective data from 1990 to 2020, suggest that healthcare spending and adolescent female fertility are essential factors in reducing maternal and neonatal mortality in South Asia. Although healthcare spending in South Asia has increased over the past decades, this was not enough for maternal and neonatal health. Our results showed healthcare spending per capita had a significant negative effect on neonatal and maternal mortality during the period under the study. The findings were consistent with an earlier study [14] that mentioned that child mortality was significantly reduced by rising healthcare expenditure. In contrast to industrialized nations, South Asian nations’ government spending on healthcare expanded more slowly than that of the developed countries’ per capita incomes [17]. Our results, like other studies, indicate that healthcare spending shows a significant negative association with infant and neonatal mortality [18].

The adult female literacy rate is one of the important indicators of reducing maternal and neonatal mortality because the literacy rate helps to increase knowledge of maternal, neonatal health and service utilization [19]. Literacy on maternal and neonatal mortality have both direct and indirect link with the availability of reproductive health care facilities. Women who educated are more likely to be rich [20, 21], have good nutrient status [22], make decisions concerning their healthcare and well-being [23], have fewer babies [24], and have accessibility to and use resources [25]. These benefits, which come from rising female literacy, reduce maternal morbidity and mortality. As female literacy rates increase, maternal death rates should go down. The modelling results of 8 countries in South Asia showed the adult female literacy rate has a significantly negative effect on neonatal and maternal mortality, which means that increasing the adult female literacy rate is vital to decreasing maternal and neonatal mortality. Similar findings have reported that maternal mortality is significantly correlated with the literacy rate [7, 26].

It is widely known that adolescent female fertility carries a higher risk of mortality. We have found a significant positive effect on the young female fertility rate in neonatal and maternal mortality. The mortality risk was highly associated with adolescent fertility because young females want to avoid early childbearing, which has social, economic, and educational consequences for young mothers [27–29]. In addition, marriage before the age of 18 has been positively linked to higher fertility, worse maternal and reproductive health, and worse health and developmental outcomes in their offspring through a variety of mechanisms involving biological elements, and maternal behaviour [30]. Globally, nearly 650 million girls and women are currently being married before their 18th birthdays [31], even though laws and human rights frameworks call for this to be prohibited. One in twenty-five of these women are being from South Asia [32]. In areas where the two are closely correlated, strengthening initiatives to delay the age at marriage may aid in reducing adolescent pregnancies.

This study pointed out that the number of healthcare providers as physicians for every 1000 people has negatively affected new-born mortality but positively affected maternal mortality. Data indicates that there was a shortage of healthcare providers in comparison with population, which might affect the quality of maternal health service provision, reproductive health, and neonatal health [33]. The previous study found the number of physicians supplied contributed to the decline in infant mortality [34]. These findings have like our results.

Our study points to several implications for future research and programs around maternal and neonatal mortality, adolescent fertility, and literacy. Further programmatic efforts should be implemented to ensure increased healthcare spending on maternal and neonatal health and increase opportunities for ongoing female education to improve literacy rates. To maximize the efficient use of healthcare spending on maternal and neonatal health, further efforts are needed from government in the South Asia regions [35]. Our findings also advocated healthcare providers’ as physicians’ being mediators to improve maternal and neonatal health [36].

The potential effects of adolescent fertility were analysed, healthcare spending was examined in relation to maternal and neonatal mortality, and the relationship between female literacy and improved knowledge of maternal, neonatal, and health care utilization was quantified among South Asian countries. This study was the most comprehensive South Asian study to date. In some South Asian regions, adolescent female fertility has increased the likelihood of maternal and new-born mortality.

### Limitations

The study is limited in the sense that some of the health outcome variables for South Asian countries did not have complete observations and have been estimated with imputation methods for the analysis of panel data. The results may be caused by the purchasing power parity process itself rather than the result of the interaction of the variables presented.

## Conclusions

The modelling results supported the theory that healthcare spending has a statistically significant effect on neonatal and maternal mortality. As a result, more attention was needed to the factors influencing neonatal and maternal mortality, as well as adolescent female fertility [37, 38]. Based on the findings, it can be noted that policymakers need to consider the importance of increasing healthcare spending to reduce neonatal and maternal mortality. The findings have further illustrated that investment in female education and avoiding adolescent fertility is indispensable for neonatal and maternal health.

## Supplementary Information

**Additional file 1: Table S1.** The definition of selected variables.

## Data Availability

The datasets used and/or analysed during this study are available from the corresponding author on reasonable request.
