# Supplementary material for: Effect of adolescent female fertility and healthcare spending on maternal and neonatal mortality in low resource setting of South Asia
Source: Health Econ Rev. 2022 Sep 17;12:47. doi: 10.1186/s13561-022-00395-7 (PMC9482740; doi:10.1186/s13561-022-00395-7)
Supplement: Supplementary file 1 — Additional file 1: Table S1. The definition of selected variables. [file 13561_2022_395_MOESM1_ESM.docx]

**Table S1**. The definition of selected variables

| **Variables** | **Definition and measure** |
| --- | --- |
| NM | Neonatal deaths per 1,000 live births |
| MM | Maternal deaths per 100,000 live births |
| HEPC | Healthcare expenditure (% of GDP), PPP |
| P | Physicians per 1,000 people |
| AFLR | Literacy rate, adult female (% of females aged 15 and above) |
| AFR | Adolescent female fertility rate (births per 1,000 women aged 15-19) |
